# *Vhl* deletion in renal epithelia causes HIF-1α-dependent, HIF-2α-independent angiogenesis and constitutive diuresis

**DOI:** 10.18632/oncotarget.11275

**Published:** 2016-08-12

**Authors:** Désirée Schönenberger, Michal Rajski, Sabine Harlander, Ian J. Frew

**Affiliations:** ^1^ Institute of Physiology, University of Zurich, Zurich, Switzerland; ^2^ Zurich Center for Integrative Human Physiology, University of Zurich, Zurich, Switzerland

**Keywords:** VHL, HIF-α, kidney, angiogenesis, diuresis

## Abstract

One of the earliest requirements for the formation of a solid tumor is the establishment of an adequate blood supply. Clear cell renal cell carcinomas (ccRCC) are highly vascularized tumors in which the earliest genetic event is most commonly the biallelic inactivation of the *VHL* tumor suppressor gene, leading to constitutive activation of the HIF-1α and HIF-2α transcription factors, which are known angiogenic factors. However it remains unclear whether either or both HIF-1α or HIF-2α stabilization in normal renal epithelial cells are necessary or sufficient for alterations in blood vessel formation. We show that renal epithelium-specific deletion of *Vhl* in mice causes increased medullary vascularization and that this phenotype is completely rescued by *Hif1a* co-deletion, but not by co-deletion of *Hif2a*. A physiological consequence of changes in the blood vessels of the *vasa recta* in *Vhl*-deficient mice is a diabetes insipidus phenotype of excretion of large amounts of highly diluted urine. This constitutive diuresis is fully compensated by increased water consumption and mice do not show any signs of dehydration, renal failure or salt wasting and blood electrolyte levels remain unchanged. Co-deletion of *Hif1a*, but not *Hif2a*, with *Vhl*, fully restored kidney morphology and function, correlating with the rescue of the vasculature. We hypothesize that the increased medullary vasculature alters salt uptake from the renal interstitium, resulting in a disruption of the osmotic gradient and impaired urinary concentration. Taken together, our study characterizes a new mouse model for a form of diabetes insipidus and non-obstructive hydronephrosis and provides new insights into the physiological and pathophysiological effects of HIF-1α stabilization on the vasculature in the kidney.

## INTRODUCTION

pVHL, the product of the von Hippel-Lindau gene (*VHL*) is a multipurpose adaptor protein that controls a wide range of biological activities including acting as the substrate recognition factor of an E3 ligase complex that targets the hypoxia-inducible transcription factor α (HIF-1α, HIF-2α and HIF-3α, collectively HIF-α) subunits for oxygen-dependent proteolysis [1]. Under hypoxic conditions, or when functional pVHL is missing, HIF-1α and HIF-2α are stabilized and induce the transcription of numerous genes that coordinate many processes including proliferation [2], erythropoiesis [3], angiogenesis [2] and metabolism [4, 5]. *VHL* is biallelically inactivated as the earliest genetic alteration in over 90% of cases of clear cell renal cell carcinoma (ccRCC), the most frequent renal malignancy. HIF-1α and HIF-2α stabilization, as well as HIF-α target genes, can be detected in *VHL*-deficient renal epithelial cells in the context of normal tubules or microscopic ccRCC precursor lesions in kidneys of patients with inherited VHL disease, indicating that constitutive HIF-α activity is present even before tumors form [6]. While numerous mouse models have shown that neither genetic deletion of *Vhl*, nor stabilization of HIF-1α and HIF-2α alone or together in renal epithelial cells are sufficient to cause tumor formation in mouse kidneys (reviewed in [7]), we have recently shown that both HIF-1α and HIF-2α are necessary for the initiation of renal cysts and tumors in *Vhl/Trp53* mutant mice [8]. Thus, HIF-1α and HIF-2α stabilization are necessary but not sufficient for renal tumorigenesis. A major experimental challenge will be the identification of which aspects of the activities of HIF-1α and HIF-2α, separately and cooperatively, are important for the initiation and progression of ccRCC. We have previously demonstrated that stabilization of HIF-1α is specifically necessary for alterations in cellular glucose metabolism, mitochondrial oxidative metabolism and mitochondrial abundance in *Vhl*-deficient and *Vhl/Trp53*-deficient renal epithelial cells *in vivo* [8], suggesting that these might be some of the cellular alterations that are required for tumour initiation.

ccRCC is an example of a highly vascularized tumour, with a typical growth pattern characterized by acinar clusters of tumour cells surrounded by an intricate network of small blood vessels. Increased blood vessel formation can already be observed surrounding microscopic *VHL* mutant ccRCC precursor lesions in kidneys of VHL patients, showing that the induction of neoangiogenesis occurs at the earliest stage of tumor development [6]. Guided by our analyses of a series of physiological phenotypes in *Vhl*-deficient mice, in this study we have investigated the consequences of tubular epithelial cell-specific stabilization of HIF-1α and HIF-2α in causing alterations to the vascular network surrounding *Vhl* mutant epithelial cells. The starting point for this study was our previous demonstration that renal epithelium-specific deletion of *Vhl* causes hydronephrosis in mice through an uncharacterized mechanism [9].

Hydronephrosis, characterized by the expansion of the renal pelvis and the collecting system, is one of the most frequently diagnosed prenatal abnormalities with an incidence of approximately 0.6% [10, 11]. While most cases of antenatal hydronephrosis spontaneously improve or resolve, 15-40% of patients require medical or surgical intervention to prevent chronic kidney disease or end stage renal disease [12]. The underlying aetiologies of prenatal hydronephrosis are diverse, such as obstructions of the urogenital tract, delayed ureteral patency during development or excess urine production [10, 13]. In adults, hydronephrosis can be induced by kidney stones, pelvic neuroblastoma, pregnancy or impaired ureteral peristaltic activity [14, 15]. Transgenic mouse models have provided insights into the molecular nature of fetal malformations, highlighting the importance of specific signalling pathways, including Hedgehog and β1-integrin, in the development of the urogenital system [16–18]. Interestingly, various knockout mouse models that display phenotypes characterized by high urinary output also exhibit a dilatation of the renal pelvis and hydronephrosis (reviewed in [19]). For instance, mutation of *Aqp2*, a mouse model of nephrogenic diabetes insipidus (NDI), causes polyuria, hydronephrosis and kidney insufficiency [20]. Similar phenotypes have been observed in mice harbouring deletions of the genes encoding Na-K-2Cl-Cotransporter (NKCC2) or the renal outer medullary potassium channel (ROMK), two models for Bartter's syndrome [21–23]. While hydronephrosis is only rarely observed in the corresponding human diseases [24, 25], the hydronephrosis phenotype is a frequent indicator of various diabetes insipidus phenotypes in mice.

Given these observations, coupled with the fact that the structure of the renal vasculature is important for ensuring correct urinary concentration, we further investigated the effects of loss of *Vhl* on urinary concentration and other renal functions that could be the cause of the hydronephrosis phenotype. We demonstrate that these mice exhibit HIF-1α-dependent defects in urinary concentration related to increased medullary vascularization, revealing HIF-1α as the angiogenic transcription factor that induces crosstalk between *Vhl*-mutant renal epithelial cells and vascular endothelia.

## RESULTS

### Loss of *Vhl* causes *Hif1a*-dependent non-obstructive hydronephrosis

**Figure 1:**
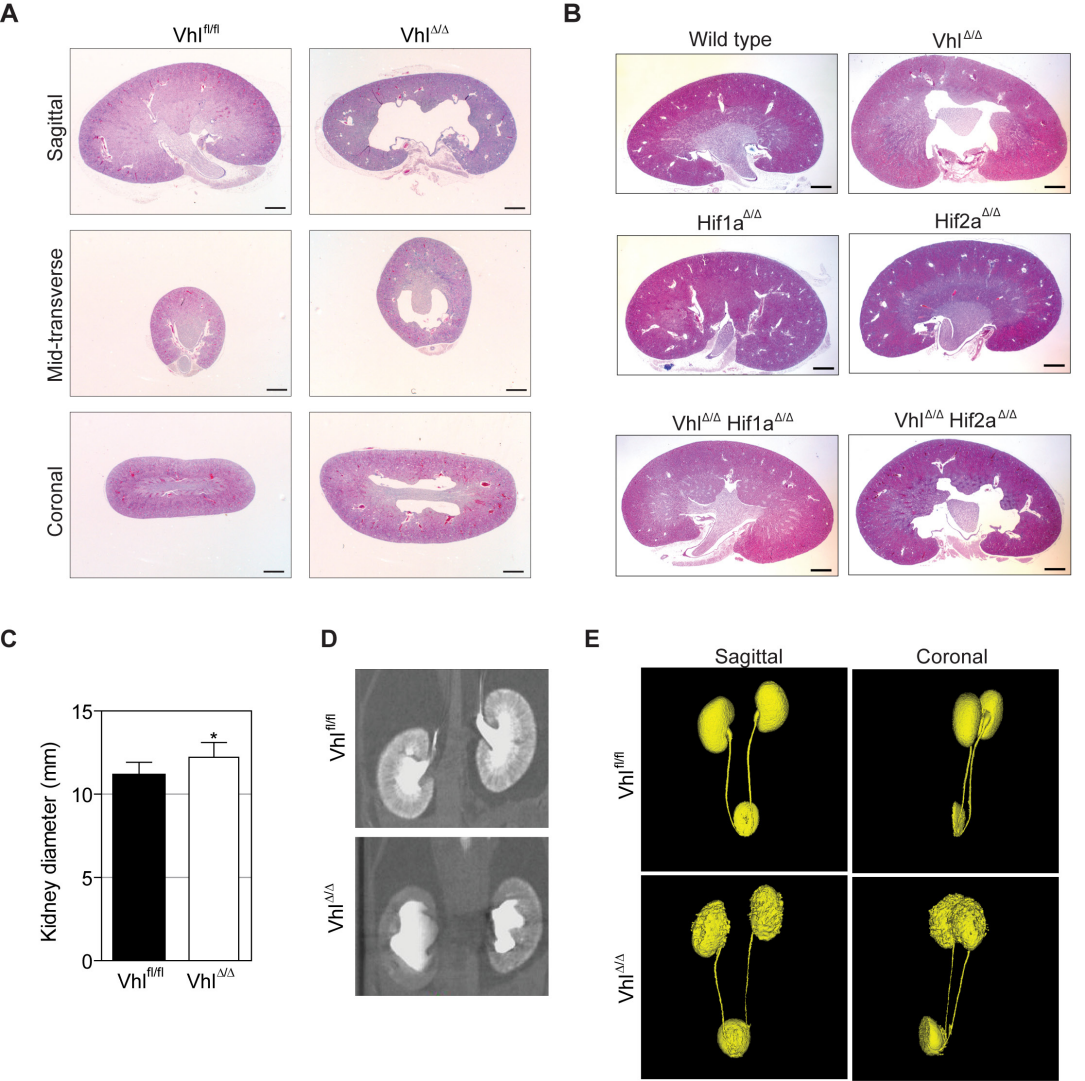
Loss of *Vhl* causes *Hif1a*-dependent non-obstructive hydronephrosis **A.** H&E stainings of different section planes of adult kidneys with a renal epithelial cell-specific deletion of *Vhl.* **B.** Overview pictures of *Vhl, Hif1a*, *Hif2a*, *Vhl/Hif1a* and *Vhl/Hif2a* deficient kidneys. **C.** Measurement of kidney size *in vivo* based on **D.** Two-dimensional μCT images of control (Vhl^fl/fl^) and *Vhl*-deficient kidneys (Vhl^Δ/Δ^). **E.** Three-dimensional reconstruction of μCT images of kidney, ureter and bladder. Scale bars = 1 mm.

Using the *Ksp1.3-Cre* driver [26] to induce gene deletion in renal epithelial cells in all segments of the nephron we recently generated renal epithelium-specific conditional knockout mice for *Vhl* [9], *Hif1a*, *Hif2a*, *Vhl/Hif1a* or *Vhl/Hif2a* [8], hereafter referred to as Vhl^Δ/Δ^, Hif1a^Δ/Δ^, Hif2a^Δ/Δ^, Vhl^Δ/Δ^Hif1a^Δ/Δ^ and Vhl^Δ/Δ^Hif2a^Δ/Δ^, respectively. *Vhl* deletion caused a morphological hydronephrosis phenotype (Figure 1A). The co-deletion of *Hif1a*, but not of *Hif2a*, completely rescued this phenotype (Figure 1B). This genetic rescue prompted further investigation of the cause of hydronephrosis following *Vhl* deletion. Given that hydronephrosis is often caused by an obstruction or dysplasia of the ureter [10, 27], we first visualized the morphology of the urinary system by micro-computed tomography (μCT) after injection of the contrast agent Visipaque, which is concentrated by the renal tubular system. Measurement of kidney size *in vivo* demonstrated a 20% increase in diameter of Vhl^Δ/Δ^ kidneys, consistent with the observed expansion of the renal pelvis in histological sections (Figure 1C). Vhl^Δ/Δ^ kidneys displayed altered structures of the medulla and papilla and the cortex typically displayed a weaker signal from the contrast agent when compared to the cortex of wild type kidneys, suggestive of changes in urine flow, concentration or renal filtration (Figure 1D). However, we did not observe a noticeable delay in renal clearance of the contrast agent and its accumulation in the bladder and 3-D rendering of the kidney volume, ureter and bladder did not show any urinary obstruction or development of hydroureter (Figure 1E). Previously published histological analyses also revealed no alterations in the urothelial cells of the renal pelvis or ureter [9].

### *Vhl*-deficient mice display *Hif1a*-dependent polyuria and hypo-osmotic urine

We utilized metabolic cages to further analyse renal handling of water, revealing that adult Vhl^Δ/Δ^ mice drink and urinate dramatically more than littermate controls (Table 1). These strong polydipsia and polyuria phenotypes are suggestive of constitutive diuresis, a conclusion supported by the finding that urine from Vhl^Δ/Δ^ mice was hypo-osmolar and was more alkaline than that of control mice. These diabetes insipidus phenotypes were completely absent in Vhl^Δ/Δ^Hif1a^Δ/Δ^ mice but present in Vhl^Δ/Δ^Hif2a^Δ/Δ^ mice, demonstrating that the constitutive stabilization of HIF-1α is the cause of constitutive diuresis. Renal epithelium-specific deletion of *Hif1a* or *Hif2a* alone did not affect diuresis. No differences in body weight, food intake or faeces production were observed in any of the knockout genotypes. These data suggest that tubular stabilization of HIF-1α causes an increased production of urine that exceeds the capacity of the renal system, likely building up a back-pressure on the kidney and inducing the formation of hydronephrosis.

**Table 1:** Characterization of feeding parameters of mice with renal epithelial cell-specific deletions of *Vhl, Hif1a, Hif2a, Vhl/Hif1a and Vhl/Hif2a.*

	Body weight (g)		Water intake (g) / body weight (g) / 24h		Urine output (g) / body weight (g) / 24h		Urine osmolality (mOsm/kg H_2_O)		Urine pH		Food intake (g) / body weight (g) / 24h		Feces (g) / body weight (g) / 24h	
**Hif1a**^fl/fl^	24.80	± 2.46	0.11	± 0.07	0.03	± 0.02	4782.50	± 1357.00	6.23	± 0.2	0.13	± 0.03	0.05	± 0.03
**Hif1a**^Δ/Δ^	25.23	± 3.82	0.12	± 0.027	0.04	± 0.01	4234.00	± 1368.60	6.17	± 0.1	0.17	± 0.07	0.05	± 0.04
**Hif2a**^fl/fl^	25.47	± 3.76	0.21	± 0.06	0.07	± 0.04	2912.00	± 958.29	6.11	± 0.17	0.19	± 0.05	0.08	± 0.03
**Hif2a**^Δ/Δ^	23.57	± 5.23	0.20	± 0.07	0.04	± 0.02	3971.90	± 1174.96	**5.82**	± 0.08*	0.21	± 0.05	0.09	± 0.03
**Vhl**^fl/fl^	31.26	± 8.90	0.17	± 0.05	0.04	± 0.04	5956.67	± 1753.58	5.94	± 0.22	0.14	± 0.04	0.06	± 0.02
**Vhl**^Δ/Δ^	27.25	± 4.94	**0.68**	± 0.23***	**0.42**	± 0.20**	**629.44**	± 315.46***	**6.92**	± 0.74***	0.16	± 0.02	0.08	± 0.03
**Vhl^fl/fl^ Hif1a^fl/fl^**	23.26	± 3.95	0.21	± 0.04	0.08	± 0.02	2896.00	± 925.86	6.26	± 0.2	0.16	± 0.02	0.06	± 0.01
**Vhl^Δ/Δ^ Hif1a^Δ/Δ^**	24.17	± 3.62	0.23	± 0.05	0.07	± 0.02	2786.00	± 412.59	6.18	± 0.2	0.17	± 0.01	0.06	± 0.01
**Vhl^fl/fl^ Hif2a^fl/fl^**	28.64	± 2.05	0.17	± 0.03	0.05	± 0.03	3313.33	± 1778.22	6.22	± 0.24	0.14	±0.019	0.06	± 0.01
**Vhl^Δ/Δ^ Hif2a^Δ/Δ^**	28.14	± 3.24	**0.65**	± 0.26**	**0.45**	± 0.20**	**410.00**	± 202.89*	**7.47**	± 0.82**	0.16	± 0.02	0.07	± 0.03

After 2 days of acclimatization, feeding parameters of mice housed in metabolic cages were measured on 5 consecutive days. Mean ± std.dev, n = 6 animals per genotype, males and females mixed, 2-3 months old. Student's unpaired t-test.

*, ** and *** denote *p* < 0.05, *p* < 0.01 and *p* < 0.001 respectively.

### Hydronephrosis in *Vhl*-deficient mice forms between postnatal days 11 and 14

**Figure 2:**
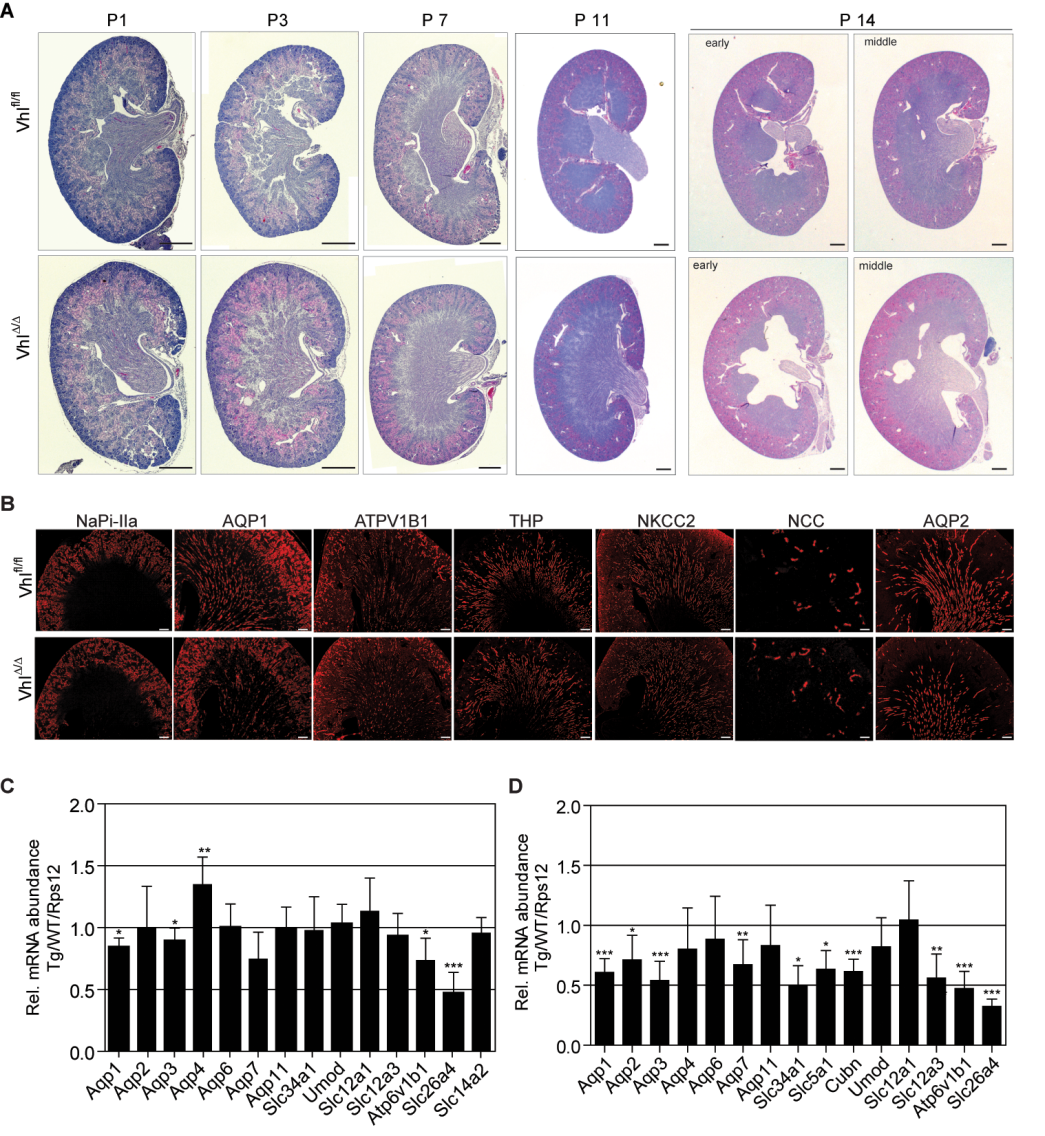
Hydronephrosis in *Vhl*-deficient mice forms between postnatal days 11 and 14 **A.** H&E stainings of *Vhl*-deficient kidneys at postnatal day 1, 3, 7, 11 and 14. Scale bars = 500 μm. **B.** Immunofluorescent staining of depicted renal proteins in kidneys of 14 day-old mice. Scale bars = 200 μm. **C.**-**D.** mRNA abundance of indicated genes in *Vhl*-deficient kidneys of (C) 11 day-old mice or (D) adults. Mean ± std.dev, *n* = 6 animals per genotype. Gene expression ratios between Ksp1.3-Cre and wild type (WT) mice, normalized to the expression of Rps12.

We next analysed early postnatal renal development to determine whether a developmental defect in the nephron may underlie these phenotypes. Comparisons of wild type and Vhl^Δ/Δ^ kidneys at postnatal days 1, 3, 7 and 11 revealed that kidney development progresses normally, with no hydronephrosis, nor gross morphological aberrations in the cortex, medulla or papilla, being observed at these ages (Figure 2A). However, kidneys of 14 day-old Vhl^Δ/Δ^ mice displayed hydronephrosis, indicating that this phenotype arises late in the second week of postnatal life. Immunofluorescence staining for markers of different nephron segments in kidneys of 14 day-old mice revealed that there are no gross alterations in nephron formation in Vhl^Δ/Δ^ kidneys (Figure 2B).

### *Vhl*-deficient kidneys express specific markers for all renal segments

We next investigated in whole kidney mRNA preparations of wild type and Vhl^Δ/Δ^ kidneys the mRNA expression levels of a range of solute and water transporters, as alterations in their expression could conceivably account for the polyuria phenotype. With the caveat that their expression may be regulated by other factors, these genes also serve as “markers” to provide a semi-quantitative assessment of the relative abundance of cells in different nephron segments before and after the onset of hydronephrosis. 11 day-old Vhl^Δ/Δ^ kidneys displayed very mild decreases of *Aqp1* (proximal tubules and descending limb of Henle), *Aqp2* (collecting ducts) and *Atp6v1b1* (intercalated cells) expression (Figure 2C). The abundance of *Slc26a4* mRNA, encoding the Cl^−^/HCO_3_^−^ exchanger Pendrin, which is expressed in the type B-intercalated cells of the cortical collecting ducts, was decreased by 50%. It is highly unlikely that reduced Pendrin expression is the primary cause of polyuria in *Vhl*-deficient mice as it has been shown that knockout of Pendrin in the mouse kidney can be compensated by NCC activity [28] and mRNA and protein expression of NCC in Vhl^Δ/Δ^ kidneys is unaltered (Figure 2B and 2C). Analysis of adult kidneys with severe hydronephrosis revealed a decrease of the mRNA abundance of genes expressed in proximal tubular cells (*Aqp1, Aqp7, Slc34a1, Slc5a1, Cubn*), distal tubular cells (*Slc12a3*), collecting duct cells (*Aqp2, Aqp3*) and intercalated cells (*Atp6v1b1, Slc26a4*), suggesting that the relative number of these cell types may be decreased in Vhl^Δ/Δ^ adult kidneys (Figure 2D). Since the expression of several proximal tubule-specific genes is decreased in adult *Vhl*-deficient mice, but the *Ksp1.3-Cre* transgene drives gene deletion in only a small percentage of proximal tubular cells, it is likely that this observation reflects progressive kidney damage and nephron loss due to persistent hydronephrosis rather than representing the primary cause of the phenotype.

### Loss of *Vhl* does not alter the proliferation capacity of renal cells *in vivo*

**Figure 3:**
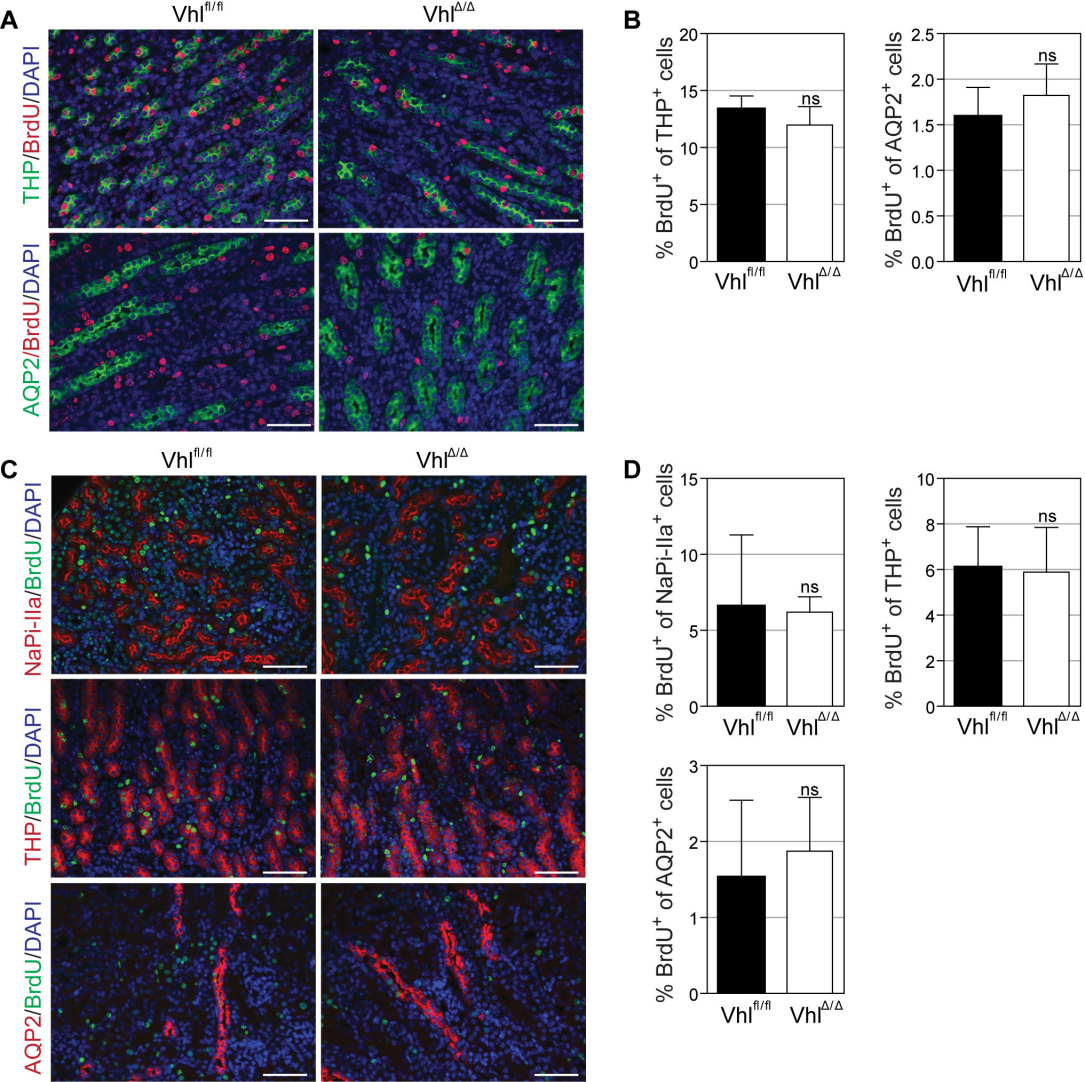
Loss of *Vhl* does not alter proliferation capacity of renal cells *in vivo* **A.**-**D.** Immunofluorescent co-staining and quantification of BrdU-labelled proliferating cells and depicted nephron markers at postnatal day 7 (A-B) or day 14 (C-D). BrdU (80 μg/g body weight) was injected intraperitoneally 2h (A) or 48h (C) before euthanasia. NaPi-IIa serves as marker for proximal tubules, Tamm-Horsfall protein (THP) for thick ascending limbs of Henle and Aquaporin 2 (AQP2) for collecting ducts. Scale bars = 50 μm, *n* = 4.

Since genetic loss of *Vhl* has previously been shown to cause senescence or reduce proliferation of various mouse cells [29–31] we further investigated whether *Vhl* deletion in highly proliferating epithelial cells may cause subtle cellular proliferative phenotypes that affect the formation of the nephron. We labelled S-phase cells in kidneys in 7 day-old mice by BrdU injection and identified thick ascending loops of Henle and collecting ducts by co-staining with antibodies against THP and AQP2, respectively. No differences in the percentage of proliferating cells in these tubule segments were observed between wild type and Vhl^Δ/Δ^ kidneys (Figure 3A-3B). Similarly, marking S-phase cells in proximal tubules (using an antibody against NaPi-IIa), thick ascending loops of Henle and collecting ducts in 14 day-old kidneys revealed no alterations in proliferation between wild type and Vhl^Δ/Δ^ kidneys (Figure 3C-3D). Taken together, we conclude that a post-natal developmental defect in the formation of the nephron is not responsible for the onset of hydronephrosis.

### Renal epithelial stabilization of HIF-1α does not impair renal electrolyte homeostasis

**Figure 4:**
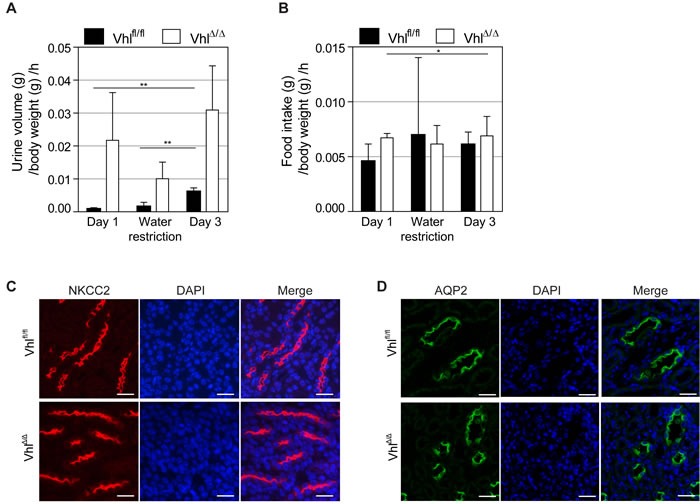
Water restriction causes severe dehydration of *Vhl*-deficient mice and there are no alterations in AQP2 or NKCC2 localization **A.** Urine volume per hour and **B.** food intake per hour of mice during water restriction test. Vhl^Δ/Δ^ mice and control littermates (Vhl^fl/fl^) were housed in metabolic cages and either provided with powder chow and water *ad libitum* for 24 h (Day 1 and Day 3) or with wet food (water restriction). Experiment was aborted after 5 h due to severe dehydration of Vhl^Δ/Δ^ mice. **C.** Immunofluoresence staining of NKCC2 (red) and DAPI (blue) in thick ascending limb of Henle of unchallenged Vhl^Δ/Δ^ mice and controls. **D.** Immunofluoresence staining of Aquaporin 2 (green) and DAPI (blue) in renal collecting duct of unchallenged Vhl^Δ/Δ^ mice and controls. Scale bars = 25 μm.

We next asked whether the increased urinary output in Vhl^Δ/Δ^ mice is associated with renal insufficiency or a salt wasting phenotype. Blood plasma levels of Na^+^, Ca^2+^, K^+^, Cl^−^, albumin, phosphorus, blood urea nitrogen (BUN) and creatinine were analysed to assess kidney function in all knockout genotypes (Table 2). Plasma of Hif1a^Δ/Δ^ mice displayed a slightly increased concentration of K^+^ whereas deletion of *Hif2a* caused slightly lower levels of phosphorus (Figure 4B), suggesting that basal renal HIF-α levels are necessary for the proper regulation of potassium and phosphate homeostasis. None of the other parameters were altered in any mice, indicating that overall renal function is not significantly impaired under normal conditions, even in Vhl^Δ/Δ^ and Vhl^Δ/Δ^Hif2a^Δ/Δ^ mice that display constitutive diuresis. Interestingly, total haemoglobin and haematocrit were slightly decreased in these two genotypes, whereas EPO plasma levels and renal *Epo* mRNA abundance remained unchanged (Supplementary Figure 1A-C). Since other mouse models with constitutive diuresis display increased haematocrit levels as a consequence of dehydration [21, 22, 32], we conclude that Vhl^Δ/Δ^ and Vhl^Δ/Δ^Hif2a^Δ/Δ^ mice are not dehydrated and can adequately compensate increased urinary output with increased water intake. The slightly decreased haematocrit in Vhl^Δ/Δ^ and Vhl^Δ/Δ^Hif2a^Δ/Δ^ mice is consistent with our demonstration of reduced renal epithelial mitochondrial content and O_2_ consumption [8] and with a recent study linking renal epithelial O_2_ consumption and O_2_ sensing by Epo-producing interstitial cells [33]. 24 hour urine collections revealed increased excretion of Na^+^, K^+^ and Ca^2+^ and a trend towards increased Mg^2+^ in Vhl^Δ/Δ^ mice (Table 3), however it is a known problem with metabolic cage analyses that the percentage recovery of urine from mice with polyuria is higher than that of normal mice (due to relatively more urine being lost in normal urinary output due to coating the collection vessels or by evaporation), biasing these measurements. To correct for this we attempted to calculate fractional excretion of these ions, however the extent of polyuria meant that for 4 out of 6 Vhl^Δ/Δ^ mice the urinary creatinine levels were below detectable limits. In the remaining 2 mice the fractional excretions of Na^+^, K^+^ and Ca^2+^ were not increased compared to wild type (Table 3). Collectively, the normal blood chemistry, the absence of increased food intake and the unchanged fractional excretion of ions argues that the hydronephrosis and constitutive diuresis phenotypes do not result from an impairment of ion handling by the kidney.

**Table 2:** Characterization of blood parameters of mice with renal epithelial cell-specific deletions of *Vhl, Hif1a, Hif2a, Vhl/Hif1a and Vhl/Hif2a.*

	Na^+^ (mmol/l)		Ca^2+^ (mmol/l)		Cl^−^ (mmol/l)		K^+^ (mmol/l)		Alb (mg/dl)		Phos (mg/dl)		BUN (g/dl)		Crea (mg/dl)		Blood pH	
**Hif1a^fl/fl^**	143.00	± 1.67	1.24	± 0.04	115.83	± 0.98	4.17	± 0.4	1.32	± 0.14	6.97	± 0.48	21.33	± 3.83	0.09	± 0.03	7.29	± 0.05
**Hif1a^Δ/Δ^**	142.00	± 1.41	1.22	± 0.02	116.17	± 1.47	4.58	± 0.58	1.42	± 0.13	**5.78**	± 0.29***	19.67	± 2.16	0.10	± 0.04	7.35	± 0.04
**Hif2a^fl/fl^**	144.00	± 1.58	1.26	± 0.03	116.20	± 0.84	3.78	± 0.15	1.42	± 0.04	6.54	± 0.65	25.00	± 4.58	0.09	± 0.02	7.26	± 0.08
**Hif2a^Δ/Δ^**	142.57	± 1.40	1.25	± 0.04	116.00	± 2.16	**4.40**	± 0.51*	1.50	± 0.07	6.91	± 0.85	23.86	± 4.06	0.07	± 0.03	7.26	± 0.06
**Vhl^fl/fl^**	146.40	± 2.70	1.35	± 0.07	115.60	± 2.30	3.52	± 0.29	1.25	± 0.06	5.85	± 0.59	16.25	± 3.30	0.12	± 0.05	7.32	± 0.08
**Vhl^Δ/Δ^**	148.83	± 1.82	1.33	± 0.05	117.17	± 3.35	3.60	± 0.39	1.30	± 0.16	7.20	± 0.86	23.67	± 6.71	0.14	± 0.04	7.37	± 0.09
**Vhl^fl/fl^ Hif1a^fl/fl^**	144.00	± 1.90	1.32	± 0.03	110.83	± 1.47	4.60	± 0.40	1.40	± 0.11	8.05	± 1.04	27.00	± 6.84	0.13	± 0.04	7.25	± 0.08
**Vhl^Δ/Δ^ Hif1a^Δ/Δ^**	144.33	± 2.58	1.29	± 0.04	111.00	± 1.67	4.48	± 0.67	1.35	± 0.10	7.82	± 0.95	25.83	± 3.54	0.10	± 0.03	7.29	± 0.08
**Vhl^fl/fl^ Hif2a^fl/fl^**	138.50	± 3.94	1.21	± 0.06	121.17	± 5.98	4.93	± 0.52	1.20	± 0.08	7.05	± 1.22	18.09	± 3.86	0.08	± 0.05	7.04	± 0.20
**Vhl^Δ/Δ^ Hif2a^Δ/Δ^**	**142.33**	± 2.94*	1.21	± 0.04	120.33	± 1.37	5.03	± 0.69	1.16	± 0.12	7.30	± 0.87	18.42	± 3.34	0.12	± 0.03	7.12	± 0.26

Analysis of kidney function parameters in the plasma of depicted mouse cohorts. Mean ± std.dev, n = 6 animals per genotype, males and females mixed, 2-3 months old. Student's unpaired t-test.

* and *** denotes *p* < 0.05 and *p* < 0.001 respectively.

**Table 3:** Urinary excretion parameters of mice with renal epithelial cell-specific deletion of *Vhl.*

	Na^+^ (μmol/24h)		K^+^ (μmol/24h)		Mg^2+^ (mg/24h)		Ca^2+^ (mg/24h)		Crea (mg/24h)		FE_Na_ (%)		FE_K_ (%)		FE_Ca_ (%)	
**Vhl^fl/fl^**	112.90	± 27.31	481.85	± 107.05	0.570	± 0.46	0.098	± 0.09	0.43	± 0.11	0.180	± 0.03	33.68	± 13.50	0.537	± 0.54
**Vhl^Δ/Δ^**	**206.12**	± 52.31**	**713.26**	± 149.12*	1.120	± 0.73	**0.36**	± 0.06**	0.65	± 0.01	0.20	± 0.07	29.48	± 6.03	1.117	± 0.12

Analysis of total urinary excretion and fractional excretion rate (FE) of specific electrolytes and creatinine in *Vhl*-deficient mice based on 24 h urine collections. Mean ± std.dev, n = 6 animals per genotype, males and females mixed, 2-3 months old. Student's unpaired t-test.

* and ** denote *p* < 0.05 and *p* < 0.01 respectively.

### Water restriction causes severe dehydration of *Vhl*-deficient mice

Polyuria with severely low urine osmolality could result either from an impairment in arginine vasopressin (AVP) function or a dysregulation of aquaporin 2 (AQP2) or/and thick-limb Na-K-2Cl cotransporter (NKCC2). Water restriction experiments aiming to physiologically stimulate AVP secretion to induce urinary concentration were complicated by the fact that Vhl^Δ/Δ^ mice became severely dehydrated after only 5 hours of water restriction, requiring the experiment to be interrupted. While the hourly amount of urine produced during the 5 hour water deprivation was reduced compared to urine production in the presence of water, Vhl^Δ/Δ^ mice still produced 6 times the amount of urine as wild type littermates, accounting for the dehydration (Figure 4A). Fluorescent stainings for NKCC2 and AQP2 in unchallenged Vhl^Δ/Δ^ mice revealed that both transporters are predominantly localized at the apical surface of cells of the thick ascending limb of Henle or collecting duct, respectively, suggestive of tonic AVP stimulation in these mice (Figure 4C-4D). It therefore appears unlikely that polyuria is caused by the inappropriate response to AVP stimulation, arguing that a morphological alteration may underlie diuresis.

### Stabilization of HIF-1α increases renal medullary vascularization

**Figure 5:**
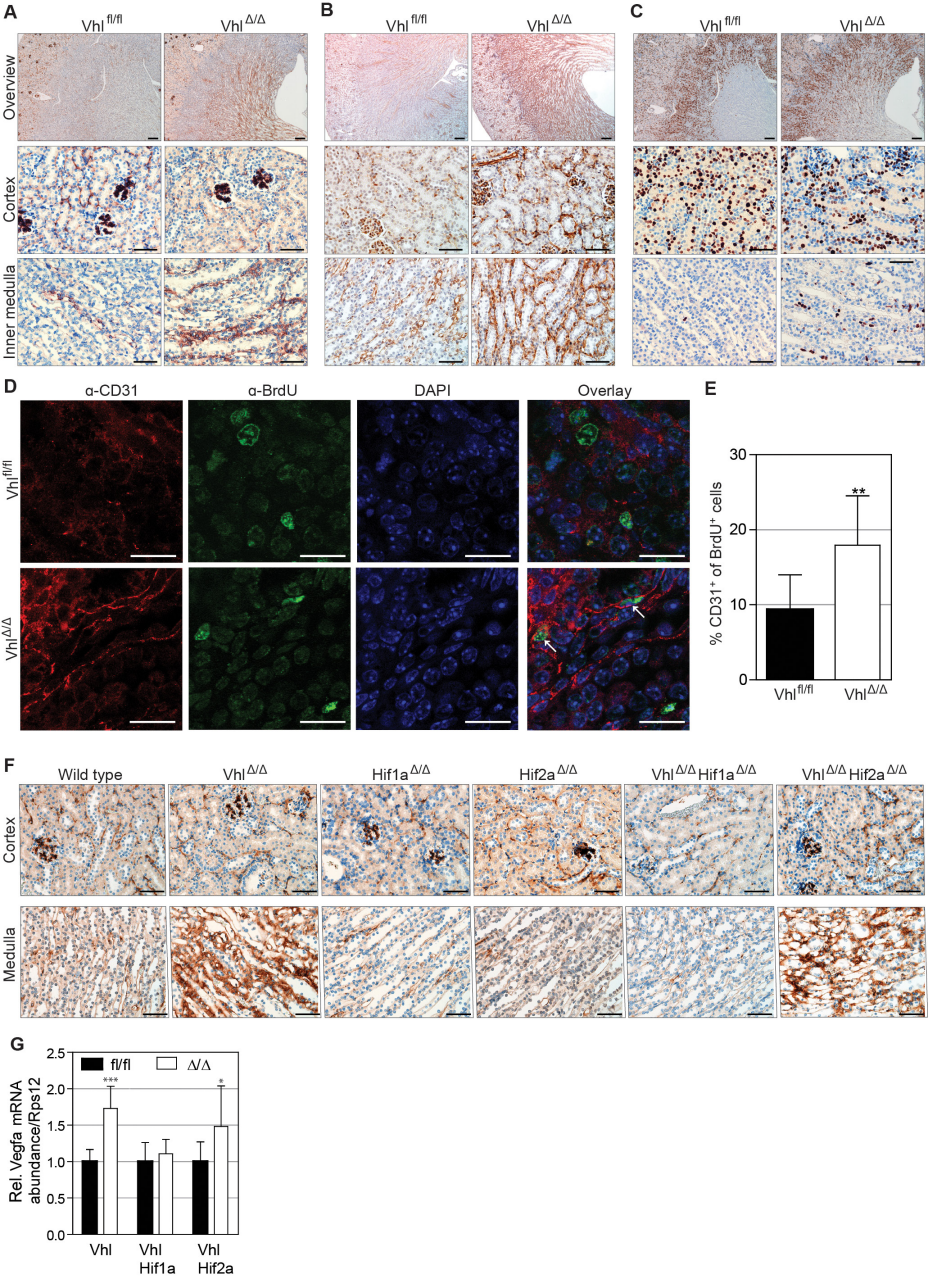
Stabilization of HIF-1α increases renal medullary vascularization Immunohistochemical staining of 14 day-old kidneys of Vhl^Δ/Δ^ and control mice (Vhl^fl/fl^) for **A.** von Willebrand Factor (vWF), **B.** CD31 and **C.** Ki67. Scale bars = 200 μm (overview images) and 50 μm, *n* = 4. **D.** Representative images and **E.** quantification of Vhl^Δ/Δ^ kidney sections and controls stained with DAPI (blue) and antibodies against BrdU (green) and CD31 (red). Scale bars = 20 μm, mean ± std.dev, *n* = 6. **F.** vWF staining on adult kidneys of depicted genotypes. Scale bars = 50 μm, *n* = 4. **G.** mRNA abundance of *Vegfa* normalized to *Rps12* in kidneys of the indicated genotypes (*n* = 6).

Since urinary concentration requires a stable tubular-interstitial concentration gradient formed by the balance of the solute pumping activities of the tubular system and removal of solutes by the blood vessels of the *vasa recta*, we examined the structure of the renal vasculature in *Vhl*-deficient mice. Kidneys from 14 day-old Vhl^Δ/Δ^ mice were stained using antibodies against von Willebrand Factor (vWF) and CD31, two markers of endothelial cells. Both antibodies revealed a striking increase in the number and density of blood vessels in the inner medulla and papilla, but not cortex, of Vhl^Δ/Δ^ kidneys (Figure 5A-5B). Staining for Ki67 to mark actively cycling cells revealed that epithelial and interstitial cells were labelled in the cortex and outer medulla in wild type and Vhl^Δ/Δ^ kidneys, whereas Ki67 almost exclusively labelled interstitial cells in the inner medulla and papilla in Vhl^Δ/Δ^ kidneys but not in wild type kidneys (Figure 5C), suggestive of increased proliferation of endothelial cells in the renal medulla. This was confirmed by co-staining for CD31 and BrdU, which showed a two-fold increase of the number of proliferating endothelial cells in the medulla of 14 day-old Vhl^Δ/Δ^ kidneys compared to controls (Figure 5D and 5E). Analyses of adult kidneys confirmed this vascular phenotype in the medulla of Vhl^Δ/Δ^ kidneys and demonstrated that this phenotype is absent in Vhl^Δ/Δ^Hif1a^Δ/Δ^ mice but present in Vhl^Δ/Δ^Hif2a^Δ/Δ^ mice (Figure 5F). Consistent with these findings, Vhl^Δ/Δ^ kidneys and Vhl^Δ/Δ^Hif2a^Δ/Δ^ kidneys, but not Vhl^Δ/Δ^Hif1a^Δ/Δ^ kidneys, displayed higher mRNA expression of the angiogenic growth factor *Vegfa* (Figure 5G). Hif1a^Δ/Δ^ mice and Hif2a^Δ/Δ^ mice displayed no observable alterations in formation of the renal vasculature. Taken together, these data support the hypothesis that HIF-1α-dependent changes in the development of the medullary vasculature may disrupt the osmotic gradient in *Vhl*-deficient mice, washing out salt from the renal interstitium und thereby preventing urine concentration.

## DISCUSSION

In this study, we aimed to identify the causes of hydronephrosis in Vhl^Δ/Δ^ mice [34] and to assess whether this phenotype relates to altered water handling by the kidney. We identified that hydronephrosis arises between postnatal day 11 and 14. μCT-based visualization of the urogenital tract in adult mice revealed no obvious delay in renal clearance of the contrast agent and an intact morphology of the ureter and the bladder, indicating that, in contrast to several other animal models for hydronephrosis [17, 35–37], *Vhl* deficient mice do not exhibit malformation or obstructions of the urogenital tract. Rather, urine production by Vhl^Δ/Δ^ mice is increased by a factor of 10 when compared to non-transgenic littermates, accompanied by a massively reduced urine osmolality, indicative of a diabetes insipidus phenotype. Although constitutive diuresis in the context of different forms of diabetes insipidus is only occasionally accompanied by hydronephrosis in humans, it is a common characteristic of different mouse models with polyuria [20–22, 38–41]. We propose that excess urine production in *Vhl*-deficient mice exceeds the ability of the ureter to remove the urine, subsequently building up pressure on the kidney, which causes expansion of the renal pelvis and the collecting system.

The hydronephrosis, polyuria and polydipsia phenotypes were completely rescued by co-deletion of *Hif1a* but not *Hif2a*, demonstrating that constitutive stabilization of HIF-1α in renal tubular cells is the cause of these phenotypes. Analyses of blood parameters in all genotypes demonstrated that renal function is not impaired and that blood electrolyte levels are maintained, despite the defects in urinary concentration. The normal food intake and ion fractional excretion rates also argue against a salt wasting phenotype and suggest that renal ion transport is not altered in *Vhl*-deficient mice. These observations are in strong contrast to most other published mouse models with polyuria, which suffer from kidney insufficiency and severe renal damage. For instance, mice suffering from NDI show progressive hydronephrosis, kidney insufficiency, growth retardation and reduced survival [20, 42]. Mouse models for Bartter syndrome, a group of genetic disorders caused by mutations in renal ion transporters, display an even more severe phenotype as they exhibit an imbalance in electrolytes in addition to the other symptoms [21, 43]. Loss *of Vhl* in the renal epithelium provides a new and unique model of diabetes insipidus in adult mice.

We show that tubular epithelial *Vhl* deletion causes increased vascular endothelial cell proliferation during the phase of postnatal renal development in which the vascular network is forming. Staining for two independent endothelial cell markers demonstrated an increased vascularization of the renal medulla in Vhl^Δ/Δ^ kidneys in both young and adult mice. These vascular phenotypes were genetically dependent on *Hif1a* but not on *Hif2a*, correlating with the rescue of the physiological urinary concentration phenotypes. The absence of vascular phenotypes in the single *Hif1a* and *Hif2a* knockout mice implies that the enhanced blood vessel formation seen in *Vhl* knockout mice is a result of an aberrant function of constitutively stabilized HIF-1α and does not reflect a requirement for normal tubular HIF-α activity as a regulator of a theoretical cross-talk between the developing renal epithelia and renal vasculature in response to a normal physiological stimulus, for example that might be due to tissue hypoxia during kidney growth.

**Figure 6:**
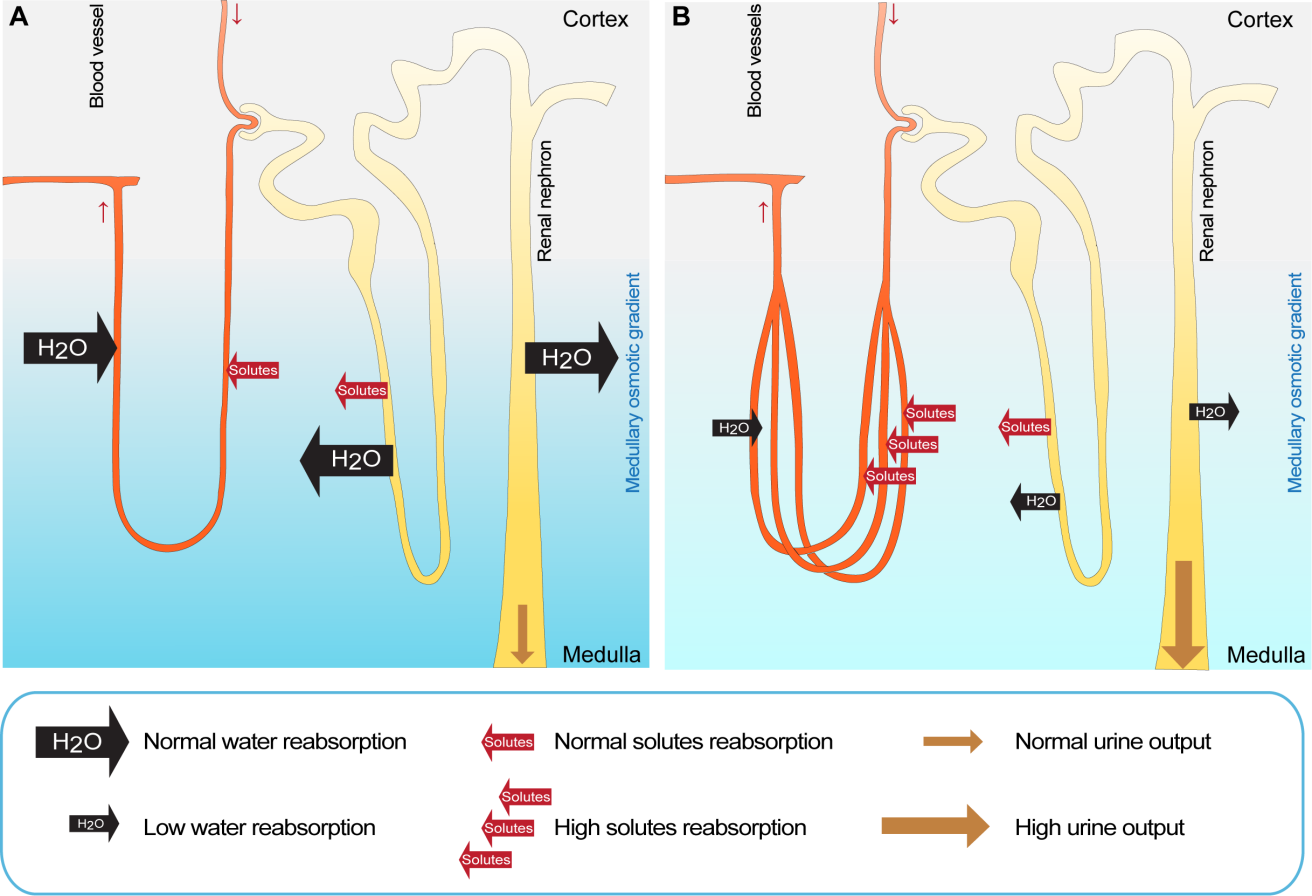
Proposed model explaining the diabetes insipidus phenotype in Vhl mice **A.** In wild type mice, renal solutes are reabsorbed from the primary urine into the interstitium by a complex network of active and passive transport that generates the medullary osmotic gradient, which is the driving force for the renal water reabsorption and uptake of solutes into the blood stream. **B.** In Vhl^Δ/Δ^ mice, the specific stabilization of HIF-1α in renal epithelial cells causes an increased vascularization of the renal medulla, leading to an elevated absorption of solutes by the blood from the renal interstitium, thereby diminishing the medullary osmotic gradient. This prevents the efficient reabsorption of water from the renal tubules and results in excess urine production. Subsequently, the urine induces a back pressure on the kidney, causing the expansion of the renal pelvis and the formation of hydronephrosis. Increased loss of water by the urinary system in Vhl^Δ/Δ^ mice is compensated by elevated water intake to prevent dehydration.

We propose that the increased renal medullary vascularization in Vhl^Δ/Δ^ mice leads to increased absorption of salts from the renal interstitium by the blood, thereby disrupting the osmotic gradient and preventing efficient urine concentration (Figure 6). The solutes that are transported across the renal tubules are therefore not lost by excretion but are efficiently returned to the blood, at the expense of urinary concentration. Consistent with this idea, several studies confirm that renal blood flow is a central mediator of ion and water absorption in the kidney. For instance, selective infusion of vasodilators in the renal medulla of rats increases papillary blood flow, accompanied by diuresis and natriuresis [44, 45]. In contrast, vasoconstrictors decreased medullary blood flow and lowered the excretion of water and sodium [46, 47]. Intriguingly, hydronephrosis has not been reported in any other model of *Vhl*-deletion in the renal epithelium, although an increase in renal vasculature has also been observed in some cases [48–52]. Given that these mice seem to exhibit a milder increase in renal vascularization than that seen in our mouse model it appears feasible to suggest that a moderate increase in renal medullary blood flow or volume, or more subtle alterations of vascular structure and vessel volume are not sufficient to dramatically disturb the renal osmotic gradient in a manner that would cause hydronephrosis. These models have however not been analyzed for their handling of water and may exhibit uncharacterized defects. We also generated inducible renal epithelial cell-specific (Ksp1.3-CreER^T2^) knockout mice for *Vhl*, however gene deletion in adult mice failed to recapitulate the urinary concentration phenotypes (Supplementary Figure 2), likely because the extent of gene deletion induced by tamoxifen was much less than achieved with the constitutive Ksp1.3-Cre driver. It is also likely that these experiments missed the important postnatal time window during which the renal vascular system develops.

Finally, our findings have important implications for understanding the development of human ccRCC as they demonstrate that HIF-1α but not HIF-2α stabilization in *Vhl* mutant “normal” mouse renal epithelial cells induces the formation of surrounding blood vessels. HIF-1α is therefore the likely factor that induces neoangiogenesis as a very early consequence of *VHL* gene mutation in human renal tubular cells, ensuring that pre-tumourigenic *VHL* mutant cells will have an adequate blood supply that will presumably support them during the earliest stages of tumour development. While it is likely that HIF-1α regulates many cellular processes that are important for tumour initiation and development, our previous observation that deletion of *Hif1a* prevents the formation of cysts and tumours in *Vhl/Trp53* mutant mice provides at least correlative evidence that is consistent with the notion that this early vascularization is important for the initiation of ccRCCs.

## MATERIALS AND METHODS

### Mouse strains

The following mouse strains were used: Ksp1.3-Cre/+; *Vhl^fl/fl^* [9] Ksp1.3-Cre/+; *Hif1a^fl/fl^*, Ksp1.3-Cre/+; *Hif2a^fl/fl^*, Ksp1.3-Cre/+; *Vhl^fl/fl^; Hif1a^fl/fl^* and Ksp1.3-Cre/+; *Vhl^fl/fl^; Hif2a^fl/fl^* [8]. Littermate mice that lacked the Cre transgene served as controls. Experiments were conducted under experimental licence 100/2013 of the Canton of Zurich.

### Immunohistochemistry

Immunohistochemistry and immunofluorescence of formalin-fixed paraffin-embedded kidneys were performed as described earlier [9, 31] using the following antibodies: α-ATPV1B1 (gift from C. Wagner), α-Aqp2 (gift from J. Loffing), BrdU (MAP3510; EMD Millipore), α-Ki67 (TEC-3; DakoCytomation), α-NaPi-IIa (gift from J. Biber), α-NCC (AB3553; EMD Millipore), α-THP (sc-20631; Santa Cruz Biotechnology) and CD31 (ab28364; Abcam), vWF (F3520; Sigma), NKCC2 (gift from J. Loffing), AQP1 (ab15080; Abcam).

### Real time PCR

RNA isolation, cDNA preparation and real time PCR were performed as described [9] using the primers listed in Supplementary Table S1.

### Measurement of feeding parameters and urine electrolytes

Single animals were adapted to the metabolic cage (Tecniplast 3600M021) for two days, followed by five measurements every 24 h. Mice were housed on a 12 h day/night cycle and provided with pulverized normal chow and water *ad libitum*. During water restriction, mice were provided with 50% powder chow in water. Urine osmolality was determined by freezing point osmometry (Osmometer, Roebling). Urine ion levels and urinary creatinine were analyzed with the UniCel^®^ DxC 800 Synchron^®^ Clinical System (Beckman Coulter). pH was measured with a pH meter from fresh urine (Seven easy, Mettler Toledo). Fractional excretion rate (FE) was calculated using the following equation ([Ion]urine/[Ion]plasma) ([Cre]urine/[Cre]plasma) [53].

### Blood collection and analysis

Blood was terminally collected from the vena cava or by puncturing the heart of isoflurane-anesthetized mice and treated with sodium-heparin. Analysis of hemoglobin and electrolyte levels, blood pH and blood gas values were performed immediately with the ABL825Flex blood gas analyzer (Radiometer). Hematocrit levels were determined by capillary centrifugation. Plasma erythropoietin levels were determined by quantitative ELISA (MEP00B, R&D Systems). Creatinine, blood urea nitrogen (BUN), albumin and phosphorus were determined using the UniCel^®^ DxC 800 Synchron^®^ Clinical System (Beckman Coulter).

### Micro-computed tomography (μCT)

μCT images were obtained with a Quantum FX microCT (Perkin Elmer) as described previously [54].

### Statistical analyses

Unless otherwise stated, data are presented as mean ± std.dev and statistical differences assessed using Student's unpaired t-test. *, ** and *** denote *p* < 0.05, *p* < 0.01 and *p* < 0.001 respectively.

## SUPPLEMENTARY MATERIALS FIGURES AND TABLE


