# Supplementary material for: Vhl deletion in renal epithelia causes HIF-1α-dependent, HIF-2α-independent angiogenesis and constitutive diuresis
Source: Oncotarget. 2016 Aug 12;7(38):60971–85. doi: 10.18632/oncotarget.11275 (PMC5308630; doi:10.18632/oncotarget.11275)
Supplement: Supplementary file 1 [file oncotarget-07-60971-s001.pdf]

Vhl deletion in renal epithelia causes HIF-1α-dependent, HIF-2α-independent angiogenesis and constitutive diuresis

Supplementary Material

A

	Hb (g/dl)	Hct (%)
**Hif1a$^{fl/fl}$**	13.98 ± 0.48	n.d.
**Hif1a$^{\Delta/\Delta}$**	14.35 ± 0.69	n.d.
**Hif2a$^{fl/fl}$**	13.14 ± 0.36	n.d.
**Hif2a$^{\Delta/\Delta}$**	13.60 ± 0.62	n.d.
**Vhl$^{fl/fl}$**	16.84 ± 0.72	50.22 ± 3.42
**Vhl$^{\Delta/\Delta}$**	**14.42 ± 0.57****	**45.60 ± 3.32***
**Vhl$^{fl/fl}$ Hif1a$^{fl/fl}$**	15.60 ± 0.60	n.d.
**Vhl$^{\Delta/\Delta}$ Hif1a$^{\Delta/\Delta}$**	15.37 ± 0.66	n.d.
**Vhl$^{fl/fl}$ Hif2a$^{fl/fl}$**	16.53 ± 0.41	50.00 ± 1.89
**Vhl$^{\Delta/\Delta}$ Hif2a$^{\Delta/\Delta}$**	**14.78 ± 1.4***	**46.33 ± 2.16***

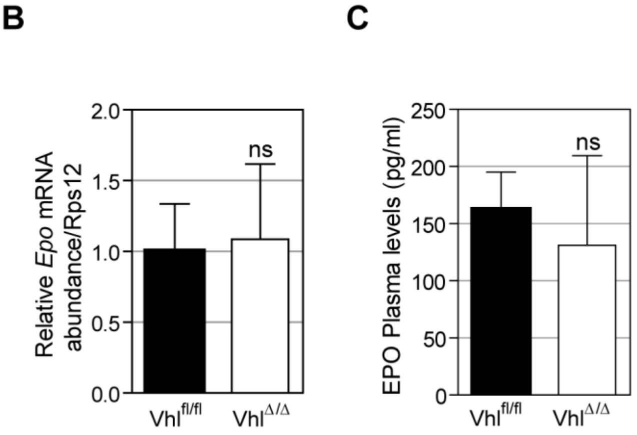

Supplementary Figure 1. Stabilization of HIF-1α decreases hemoglobin and hematocrit levels.

(A) Analysis of blood samples of *Vhl*, *Hif1a*, *Hif2a*, *Vhl/Hif1a* and *Vhl/Hif2a* deficient mice shows a HIF-1α-dependent decrease of hemoglobin and hematocrit levels with unchanged abundance of (B) renal erythropoietin (Epo) mRNA transcripts and (C) plasma EPO.

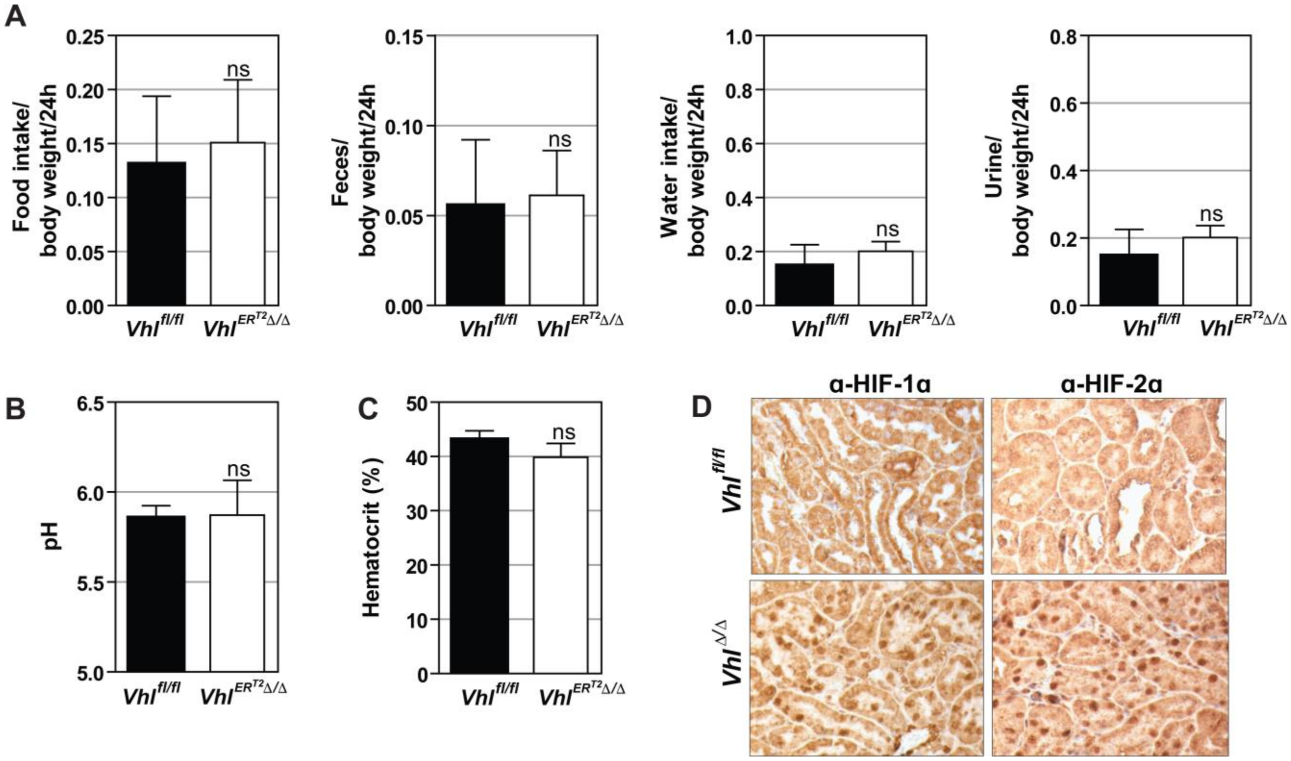

Supplementary Figure 2. Inducible deletion of *Vhl* in adult renal epithelial cells does not recapitulate the phenotype of constitutive deletion of *Vhl*.

Cre activity was induced in Ksp1.3-CreER^{T2} mice by injection of tamoxifen at age 8 weeks. (A) Food intake, faeces output, water intake and urine output, (B) Urine pH and (C) hematocrit of control Vhl$^{fl/fl}$ and inducibly-knocked out (Vhl$^{ERT2\ \Delta/\Delta}$) mice 2 months after induction of gene deletion. (D) Immunohistochemical detection of HIF-1α and HIF-2α confirming *Vhl* gene deletion. The extent of gene deletion in terms of the number of cells per kidney section displaying nuclear HIF-1α and HIF-2α was much less than seen when using the non-inducible Ksp1.3-Cre driver to effect gene deletion of *Vhl*.

Table S1: Primers used for real time PCR analyses

**Gene (primer)**	**Oligonucleotide sequence (5´-3´)**
Aqp1 (fwd)	AGG CTT CAA TTA CCC ACT GGA
Aqp1 (rev)	GTG AGC ACC GCT GAT GTGA
Aqp11 (fwd)	TGG GGC TAA TGC TGC TGT TC
Aqp11 (rev)	CAC CCA TTT CGG GG GAC ATA
Aqp2 (fwd)	ATG TGG GAA CTC CGG TCC ATA
Aqp2 (rev)	ACG GCA ATC TGG AGC ACAG
Aqp3 (fwd)	GCT TTT GGC TTC GCT GTCAC
Aqp3 (rev)	TAG ATG GGC AGC TTG ATC CAG
Aqp4 (fwd)	CTTT CTG GAA GGC AGT CTC AG
Aqp4 (rev)	CCA CAC CGA GCA AAA CAAA GAT
Aqp6 (fwd)	GTG TAG CAG GGC TTA CCT TCT
Aqp6 (rev)	GAT GGC GAT CTG GAG CAC A
Aqp7 (fwd)	AAT ATG GTG CGA GAG TTT CTG G
Aqp7 (rev)	ACC CAA GTT GAC ACC GAG ATA
Atp6v1b1 (fwd)	AAG TTT GCC CAG TAT GCT GAG
Atp6v1b1 (rev)	GCA GGA TGT CCC CTG TGA A
Cubulin (fwd)	CAC TTT AGG TTG TGG TGG AAC A
Cubulin (rev)	TTG CTG TCA AAG CTA ATC TCC C
Epo (fwd)	ACT CTC CTT GCT ACT GAT TCC T
Epo (rev)	ATC GTG ACA TTT TCT GCC TCC
S12 (fwd)	GAA GCT GCC AAA GCC TTA GA
S12 (rev)	AAC TGC AAC CAA CCA CCT TC
Slc12a1 (fwd)	TGG GTT GTC AAC TTC TGC AAT
Slc12a1 rev	CCG GGA AAT CAG GTA GTA GGC
Slc12a3 (fwd)	ACA CGG CAG CAC CTT ATA CAT
Slc12a3 (rev)	GAG GAA TGA ATG CAG GTCA GC
Slc26a4 (fwd)	AAG AGA GCC TTT GGT GTG GTA
Slc26a4 (rev)	CAG GGC ATA AGC CAT CCC TTG
Slc34a1 (fwd)	TGC CTC TGA TGC TGG CTTT C
Slc34a1 fwd	TTG TGG GTT GCC AAC ATG ATG
Slc5a1 (fwd)	ATG CGG CTG ACA TCT CAG TC
Slc5a1 (rev)	ACC AAG GCG TTC CAT TCA AAG
Umod (fwd)	CCT GGG ACA TGC AGG AAC AC
Umod (rev)	GTA GGA GAC CAC TCT GAG CCT
